# *BelloStage™-3000* Bioreactor Versus Conventional Cultivation of Recombinant Capripoxvirus Expressing *Brucella* Antigens in Vero Cells: A Step Towards the Development of a New Human Brucellosis Vaccine

**DOI:** 10.3390/cells14201631

**Published:** 2025-10-20

**Authors:** Zhanat Amanova, Zhanna Sametova, Olga Chervyakova, Sholpan Turyskeldi, Alina Kurmasheva, Ruslan Abitayev, Abdurakhman Ussembay, Zhanat Kondibayeva, Dariya Toktyrova, Dana Mazbayeva, Yerbol Bulatov

**Affiliations:** Research Institute for Biological Safety Problems, National Holding “QazBioPharm”, Gvardeiskiy 080409, Kazakhstan; zh.sametova@biosafety.kz (Z.S.); o.chervyakova@biosafety.kz (O.C.); sh.smankizi@biosafety.kz (S.T.); a.kurmasheva@biosafety.kz (A.K.); r.abitaev@biosafety.kz (R.A.); a.ussenbay@biosafety.kz (A.U.); zh.kondybaeva@biosafety.kz (Z.K.); d.toktirova@biosafety.kz (D.T.); d.mazbayeva@biosafety.kz (D.M.)

**Keywords:** capripoxvirus, brucellosis, recombinant vector, bioreactor, Vero cell line, serum-free culture medium, macrocarrier

## Abstract

**Highlights:**

**What are the main findings?**

- The BelloStage™-3000 bioreactor with BioNOC II^®^ macrocarriers enabled a ~100-fold increase in Vero cell density compared with static flasks and supported high titers of recombinant capripoxviruses.
- Maximum viral yields in the bioreactor reached up to 7.75 log_10_ TCID_50_/mL, significantly exceeding those obtained by conventional cultivation.

**What is the implication of the main finding?**

- The system represents a scalable, serum-free platform that facilitates seamless transfer of laboratory protocols to industrial production, reducing regulatory barriers and simplifying downstream processing.
- These features highlight its suitability for cost-effective vaccine manufacturing in low- and middle-income countries, where robustness and affordability are critical.

**Abstract:**

Brucellosis remains one of the most significant zoonotic diseases, posing a serious threat to both human health and livestock. This issue is particularly relevant for Kazakhstan, which is among the countries endemic for brucellosis with a high incidence rate. Such circumstances highlight the urgent need for the development and implementation of effective preventive measures, including modern vaccine platforms capable of providing reliable protection for the population and reducing the economic impact on the agricultural sector. Recombinant capripoxviruses are considered promising vector platforms for vaccine development, as they ensure high expression of target antigens, elicit strong immune responses, and are safe for humans. In this study, the replication of recombinant capripoxviruses expressing *Brucella* antigens (SPPV (TK-) OMP19/SODC and SPPV (TK-) OMP25) was evaluated in Vero cells using the BelloStage™-3000 bioreactor system in combination with BioNOC II^®^ macrocarriers. Application of the bioreactor resulted in nearly a 100-fold increase in Vero cell density compared with static cultures and provided optimal conditions for cell adhesion, growth, and metabolic activity. Consequently, a significant increase in viral titers was observed: for SPPV (TK-) OMP19/SODC, mean titers reached 7.50 log_10_ TCID_50_/mL versus 4.50 in static culture (*p* < 0.0001), while SPPV (TK-) OMP25 achieved 7.08 log_10_ TCID_50_/mL versus 4.33 (*p* < 0.001). These findings confirm the reliability, reproducibility, and scalability of this bioreactor-based approach, demonstrating clear advantages over conventional cultivation methods. Overall, the study highlights the high potential of the BelloStage™-3000 system with BioNOC II^®^ macrocarriers for the industrial production of recombinant capripoxvirus-based vaccines against brucellosis and for the broader development of other recombinant viral vaccines.

## 1. Introduction

Human brucellosis is caused by Gram-negative coccobacillus bacteria of the *Brucella* genus [1,2,3]. The present classification of his genus comprises 13 species, with the possibility of further species being counted in the future [4,5,6]. Among these, B. melitensis causes the most severe cases of brucellosis in humans and is the most common worldwide, followed by B. abortus, B. suis and B. canis [7,8,9,10]. Despite being one of the most prevalent zoonoses worldwide, attention given to global brucellosis control and prevention has been inadequate. In regions where the disease is endemic, brucellosis has far-reaching and deleterious effects on humans and animals alike [11,12,13]. The problem is especially acute in Kazakhstan, which is among the 25 countries with the highest burden of brucellosis: about 2500–3500 new cases of the disease among humans are registered here annually [14]. Under the conditions of an unfavorable epidemiological situation with brucellosis in Kazakhstan, the elimination of infection among farm animals remains the key element in preventing the disease in humans [15]. Vaccination remains the main tool in brucellosis control: live vaccines against *Brucella* abortus (S19, RB51) are used for cattle, while the *Brucella* melitensis Rev.1 vaccine is applied in small ruminants. Mass immunization reduces the prevalence of infection in livestock and interrupts the chain of pathogen transmission. However, the use of live vaccines has several limitations, including post-vaccination seropositivity, the risk of infection among personnel, and contraindications for use in pregnant animals [16]. These factors make it necessary to find new solutions. One of the promising directions is the development of recombinant vaccines based on vectors that do not possess pathogenicity for humans and animals [17,18,19,20,21,22,23,24,25]. A vector vaccine against cattle brucellosis has already been developed and registered in Kazakhstan. It is based on recombinant influenza A viruses engineered to express the *Brucella* ribosomal protein L7/L12 or the outer membrane protein Omp16, inserted into the NS1 open reading frame [26,27]. Furthermore, a similar vector vaccine is currently being developed for the prevention of brucellosis in humans, opening new perspectives for the successful implementation of the One Health concept. Despite the progress achieved in the development of vector vaccines against brucellosis, the most important task remains the creation of effective and economically affordable technologies for the production of such preparations. The large-scale application of recombinant viral vaccines is impossible without the preliminary optimization of their cultivation processes under controlled conditions. To address these challenges, laboratory-scale bioreactor models are widely used, allowing the testing of new biotechnological approaches under conditions close to industrial ones. One such system is the BelloStage™-3000 (Esco Micro Pte. Ltd., Singapore), which operates on the principle of Tide Motion. This technology provides gentle mixing, efficient gas exchange, and high cell culture viability, creating favorable conditions for enhancing viral replication under serum-free conditions. In this regard, the use of the BelloStage™-3000 system is of particular interest for enhancing the replication of recombinant capripoxvirus expressing *Brucella* antigens. Optimization of the process in this model may become an important step toward the development of scalable production technologies and bring closer the practical implementation of the concept of creating a new human anti-brucellosis vaccine.

The aim of this study was to enhance the replication of recombinant capripoxviruses expressing *Brucella* antigens in Vero cells by employing the BelloStage™-3000 laboratory bioreactor as a model of scalable serum-free cultivation and to compare its efficiency with that of conventional static culture methods.

## 2. Materials and Methods

### 2.1. Cell Lines and Culture Media

The Vero cell line, obtained from the Laboratory of Cell Biotechnology at the Research Institute for Biological Safety Problems, Republic of Kazakhstan, was used as a substrate for the propagation of recombinant capripoxviruses (SPPV (TK-) OMP19/SODC and SPPV (TK-) OMP25) and for microtitration assays. Prior to use, the Vero cells were tested and confirmed to be free of mycoplasmas. Cells were maintained in OptiPRO™ SFM medium (Gibco^™^, Life Technologies, Grand Island, NY, USA, #12309019) supplemented with 100 U/mL penicillin, 100 µg/mL streptomycin, and L-glutamine (Gibco^™^, Life Technologies, Grand Island, NY, USA, #25030081).

Mycoplasma testing was performed as described in Supplementary Materials (Supplementary File S1).

### 2.2. Virus

Recombinant capripoxviruses SPPV(TKΔ)-EGFP, SPPV(TKΔ)-OMP25, and SPPV(TK-)OMP19/SODC were obtained by homologous recombination under conditions of temporal dominant selection [17]. The plasmids pIN-TK-EGFP, pIN-TK-OMP25 [28] and pIN-TK- OMP19/SODC were used to integrate foreign genes. The pIN-TK- OMP19/SODC was derived from the basic integration plasmid [28] by cloning a chimeric sequence encoding proteins of *Brucella* spp. Cu-Zn superoxide dismutase C and lipoprotein OMP19. The sequences encoding the target proteins were spliced by overlap extension PCR with inclusion of the S2G4S2 bridge. Recombinant SPPV(TK-)-EGFP virus served as a model virus for biological activity evaluation.

Viral inocula for this study were prepared in adherent Vero cells. Briefly, Vero cells were infected with SPPV (TK-) OMP19/SODC and SPPV (TK-) OMP25 from initial amplification at a multiplicity of infection (MOI) of 0.1. Supernatants were collected 96 h post-infection, aliquoted, and stored at −40 °C. For each experiment, a fresh aliquot of the viral stock was used to avoid repeated freeze–thaw cycles.

### 2.3. Vero Cell Cultivation in Culture Flasks

Vero cells were pre-cultured in 50 mL of OptiPRO™ SFM supplemented with 1% L-glutamine in culture flasks (Techno Plastic Products AG, Trasadingen, Switzerland, #90300). For passaging, the monolayer was washed twice with phosphate-buffered saline (PBS) (Gibco, Life Technologies, Grand Island, NY, USA, #10010023), then 0.25% trypsin-EDTA (1X) (Gibco, Life Technologies, Grand Island, NY, USA, #25200056) was added. Cells were incubated at 37 °C for 2–3 min to detach. Detached cells were centrifuged at 180× *g* for 5 min, resuspended in fresh OptiPRO™ SFM, and seeded into new vials for further culture.

Static cultures were maintained in a humidified incubator at 37 °C with 5% CO_2_.

### 2.4. Virus Infection and Propagation in Culture Flasks

Flasks containing Vero cells were washed twice with PBS (20 mL per flask), and 50 mL of SPPV (TK-) OMP19/SODC or SPPV (TK-) OMP25 pre-diluted (MOI 0.1) in serum-free OptiPRO™ medium was added to each flask. Cells were incubated with SPPV (TK-) OMP19/SODC or SPPV (TK-) OMP25 inoculum for 1 h in an incubator at 37 °C with 5% CO_2_. After the adsorption period, the inoculum was removed, and 50 mL of OptiPRO™ SFM supplemented with 1% L-glutamine, 100 U/mL penicillin, and 100 µg/mL streptomycin was added. The cells were incubated under standard conditions for 5 days, with the medium replaced after 48 h, after which the supernatant was collected.

### 2.5. Vero Cell Cultivation in the BelloStage™-3000 Bioreactor

Vero cells were expanded in OptiPRO™ SFM with L-glutamine, detached with trypsin-EDTA, and inoculated (≥1.5 × 10^7^ cells/mL) into BelloCell™ 500A flasks with BioNOC II^®^ macrocarriers. After 5 h incubation at 37 °C/5% CO_2_ with gentle manual mixing, 380 mL fresh medium was added, and cultures were transferred to the BelloStage™-3000 system. Cells were grown under standard bioreactor conditions (rise/fall rate 1.0 mm/s, 10 s hold) with medium change after 48 h; cell density, pH (7.0–7.4), and glucose (>1.0 g/L) were monitored daily.

A detailed description of the Vero cell cultivation protocol using the BelloStage™-3000 system is available in the Supplementary Material File S2.

### 2.6. Viruses’ Infection and Multiplication on BioNOC II^®^ Macrocarriers

SPPV (TK-) OMP19/SODC and SPPV (TK-) OMP25 were propagated on BioNOC II^®^ macrocarriers at a seed cell density of 1.5–3.0 × 10^8^ cells. Vero cells were pre-washed with 500 mL of serum-free OptiPRO™ SFM medium, and 500 mL of inoculum (SPPV (TK-) OMP19/SODC or SPPV (TK-) OMP25) at a dose of 0.1 TCID_50_/mL was added. The BelloCell 500A culture bottle was then placed in the BelloStage™-3000 and incubated for 1 h at 37 °C with 5% CO_2_ under the following conditions: rise rate—2.0 mm/s, T_H—20 s, fall rate—2.0 mm/s, B_H—0 s. After this period, the BelloStage™-3000 parameters were adjusted to a lifting speed of 1.0 mm/s, T_H—10 s, lowering speed—1.0 mm/s, B_H—10 s. Daily samples were collected to monitor medium pH (7.2–7.4) and glucose concentration (>1.0 g/L).

### 2.7. Determination of Infectious Activity of Viruses

The infectious activity of viral samples was determined by titration in Vero cell cultures. Cells were seeded into 96-well plates (10,000 cells/well), and the following day, tenfold serial dilutions of viral samples (10^−^^1^–10^−8^) were added, with four wells per dilution. Incubation was carried out in a CO_2_ incubator at (37 ± 0.5) °C in a 5% CO_2_ atmosphere for 7 days. The cytopathic effect was assessed using a light microscope. In the case of GFP-recombinants (SPPV(TK–)-EGFP), green fluorescence of the cells was additionally recorded under a fluorescence microscope. The infectious titer was expressed as log_10_ TCID_50_/mL, calculated by the Reed–Muench method [29]. GFP expression was assessed in control cells infected with the recombinant virus SPPV(TKΔ)-EGFP. This construct contains the EGFP gene, allowing visual monitoring of infection and replication. Recombinant viruses SPPV(TKΔ)-OMP25 and SPPV(TK-)-OMP19/SODC were generated using the same viral backbone and homologous recombination strategy but contained foreign gene inserts without a fluorescent marker.

### 2.8. Assessment of Cell Density on BioNOC II^®^ Macrocarriers

Cell density was determined using the method described in reference [30]. Briefly, cell nuclei were released by placing two carriers into 1 mL of Crystal Violet (CDV) Nuclear Counting Kit (Esco Micro Pte. Ltd., Singapore, Cat. #1400014), incubating at 37 °C with intermittent shaking, and 20 µL of the resulting suspension was used for counting on a TC20 automated cell counter.

### 2.9. Glucose Level Monitoring

Glucose levels were monitored using the GlucCell™ system (Esco Micro Pte. Ltd., Singapore). Whenever the concentration in the Vero cell growth medium dropped below 1 g/L, glucose (Sigma-Aldrich, St. Louis, MO, USA, #G8769-100ML) was supplemented to 3 g/L to maintain optimal conditions for viral replication.

### 2.10. Monitoring of pH

pH was measured using a pH meter as described in [30]. If a decrease in the culture medium pH was observed, it was corrected by adjusting the CO_2_ concentration in the incubator and by periodically adding 7.0% NaHCO_3_ solution (Sigma-Aldrich, St. Louis, MO, USA, #144558).

### 2.11. Statistical Analysis of Data

Statistical analysis was performed using GraphPad Prism software version 8.0.1. Comparison of SPPV (TK-) OMP19/SODC and SPPV (TK-) OMP25 titers obtained by different cultivation methods was conducted using a two-sided Student *t*-test. Differences were considered statistically significant at *p* < 0.05.

## 3. Results

### 3.1. Vero Cell Growth and Recombinant Capripoxvirus Replication in Static Flasks

**Figure 1:**
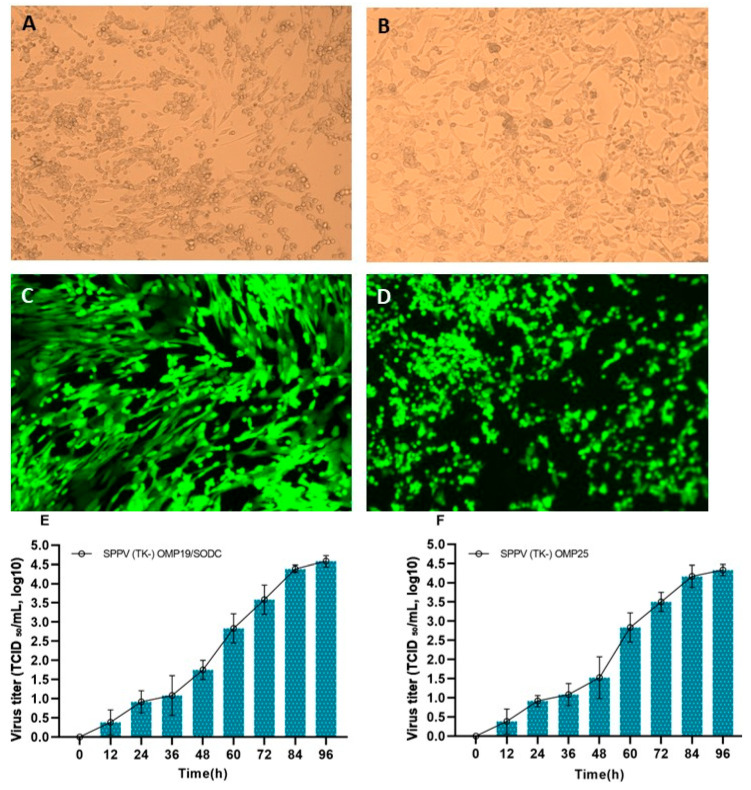
Morphology of Vero cells infected with recombinant SPPV (TK-) OMP19/SODC and SPPV (TK-) OMP25, with characteristic GFP fluorescence. Morphology of Vero cells infected with recombinant SPPV (TK-) OMP19/SODC (**A**) and SPPV (TK-) OMP25 (**B**). Showing GFP expression in control cells (**C**,**D**) as a marker confirming that the infection and replication processes were functional. Virus titers of recombinant capripoxviruses SPPV (TK-) OMP19/SODC (**E**) and SPPV (TK-) OMP25 (**F**) in Vero cell culture.

Vero cells were cultured in flasks, and their attachment and growth were monitored by light microscopy. Cells were seeded at a density of 1.5 × 10^7^ cells/mL. The results showed that Vero cells began to adhere to the flask surface within 6–8 h post-seeding, and by 24 h, the surface was fully covered. The cultured Vero cells were then infected with recombinant SPPV(TK-)-OMP19/SODC or SPPV(TK-)-OMP25 at a dose of 0.1 TCID_50_/well. On day 4 post-infection, when 80–90% of the cell monolayer was affected by the recombinant viruses (Figure 1A,B), viral suspensions were collected. GFP expression was observed in control cells infected with a GFP-expressing SPPV construct (Figure 1C,D), confirming that the infection and replication processes were functional. At this point, the maximum mean titers of SPPV(TK-)-OMP19/SODC and SPPV(TK-)-OMP25 reached 4.67 ± 0.08 log_10_ TCID_50_/mL and 4.33 ± 0.17 log_10_ TCID_50_/mL, respectively (Figure 1E,F).

### 3.2. Growth of Vero Cells in the BelloStage™-3000 Bioreactor

**Figure 2:**
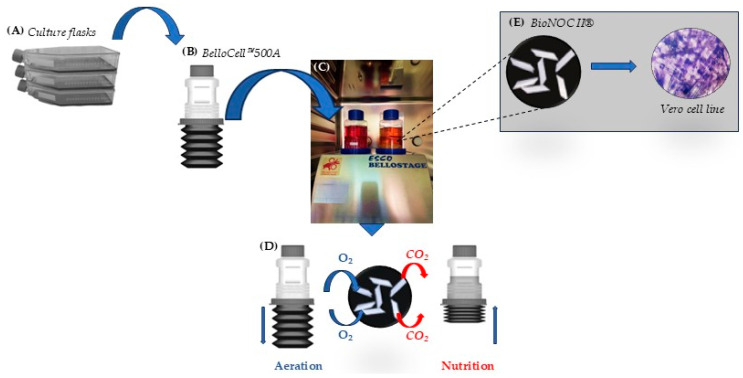
Schematic representation of the BelloStage™-3000 bioreactor system. The system was placed in a 37 °C, 5% CO_2_ incubator. The process began with the preparation and dissociation of Vero cells in OptiPRO™ SFM medium (**A**). The prepared cell suspensions were seeded into BelloCell™ 500A flasks containing BioNOC II^®^ macrocarriers (**B**). After the initial attachment phase, additional medium was added, and the flasks were transferred to the bioreactor (**C**). Cyclic up-and-down movement of the platform facilitated nutrient and O_2_/CO_2_ exchange (**D**), creating optimal conditions for Vero cells and subsequent virus replication (**E**).

The seeding cell concentration was 2.0 × 10^8^ cells. After a 5-h incubation of the BelloCell 500A flasks in an inverted position at 37 °C in a CO_2_ incubator, the attachment efficiency of Vero cells on BioNOC Ⅱ™ macrocarriers reached 98%. After cell adsorption, 380 mL of OptiPRO™ SFM medium supplemented with 1% L-glutamine was added to each BelloCell 500A flask. The white caps were then replaced with blue caps, and the flasks were installed in a BelloStage™-3000 bioreactor (ESCO BIOENGINEERING Co., Ltd., Taichung, Taiwan) for subsequent cultivation (Figure 2).

**Figure 3:**
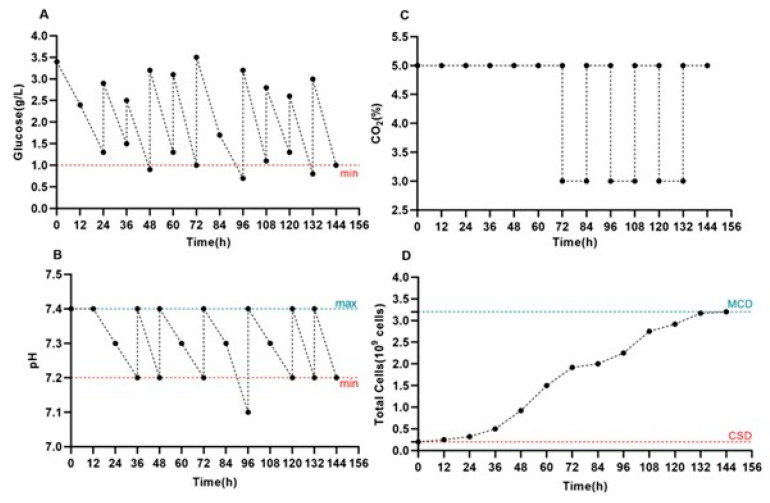
Monitoring of metabolic parameters and Vero cell growth in the BelloStage™-3000 system. Dynamics of glucose (**A**), pH (**B**), and CO_2_ (**C**) during Vero cell cultivation in the BelloStage™-3000 system with BioNOC II^®^ macrocarriers. (**D**) Cell growth kinetics are shown at 12 h intervals. (max/min). The upper and lower tolerance limits are indicated by the blue and red dotted lines, respectively. (MCD) Maximum cell density. (CSD) Cell seeding density.

The glucose content and pH of the culture medium were monitored and adjusted daily, maintaining the glucose concentration at 1–1.5 g/L (Figure 3A) and the pH within the range of 7.0–7.4 (Figure 3B). The CO_2_ level was regulated in response to the measured pH value (Figure 3C). On day 6 of culturing, the total number of Vero cells reached 3.2 × 10^9^. The growth kinetics of Vero cells on BioNOC II™ macrocarriers were determined at 12-h intervals after the attachment stage (Figure 3D).

### 3.3. Propagation of Recombinant Capripoxviruses on BioNOC II^®^ Macrocarriers

**Figure 4:**
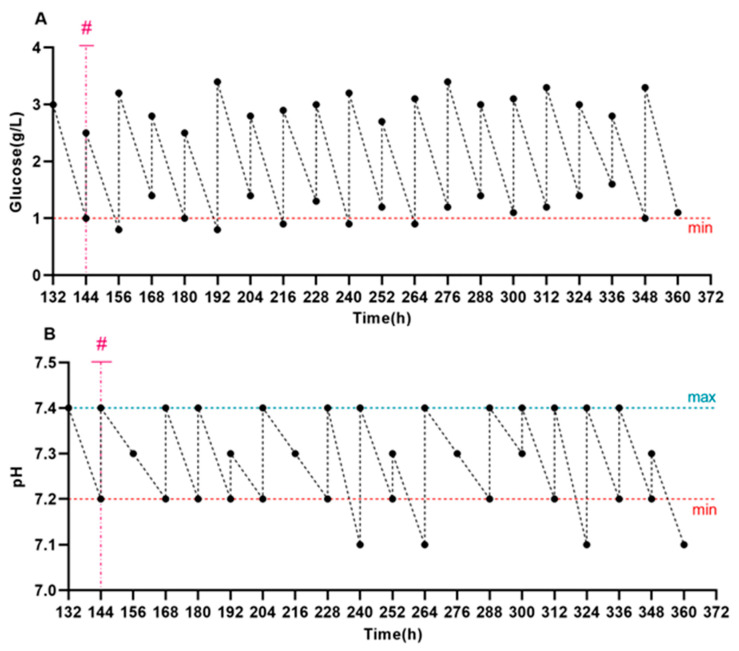

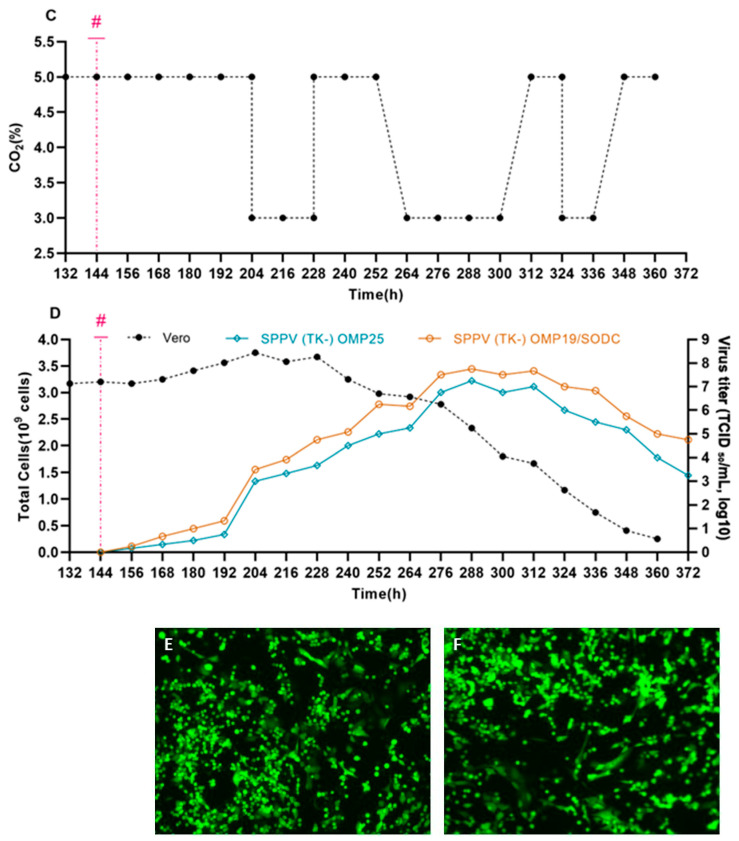
Vero cell growth and replication of recombinant SPPV with metabolic parameters monitored in the BelloStage™-3000. (**A**) Kinetics of glucose consumption. (**B**) Kinetics of pH change. (**C**) CO_2_ concentration during cultivation. (**D**) The left *y*-axis shows the total number of cells, and the right *y*-axis shows the titers of SPPV (TK-) OMP19/SODC and SPPV (TK-) OMP25 (TCID_50_/mL, log_10_). GFP expression was observed in control cells (**E**,**F**) as a marker confirming that the infection and replication processes were functional. (max/min). The upper and lower tolerance limits are indicated by blue and red dashed lines, respectively. (#) The time of infection is indicated by the magenta dashed line.

On day 6 after seeding, Vero cells were inoculated with recombinant capripoxviruses (SPPV (TK-) OMP19/SODC or SPPV (TK-) OMP25) at a dose of 0.1 TCID_50_/well. At the time of inoculation and every 48 h thereafter, the culture medium was replaced with fresh medium, and the viral suspension was collected simultaneously. The glucose concentration and pH in the culture medium were monitored daily and adjusted when necessary to ensure stable conditions: glucose was maintained at ≥1.0 g/L (Figure 4A) and pH within the range of 7.2–7.4 (Figure 4B). The CO_2_ level was regulated according to the measured pH value (Figure 4C). Cell density was monitored every 12 h (Figure 4D).

Virus cultivation was terminated after a marked decline in cell density. Monitoring results indicated that a gradual reduction in cell density began 48 h after virus inoculation. The virus was cultured for 9 days post-infection. Maximum mean virus titers were recorded on day 6 post-infection, reaching 7.67 ± 0.08 log_10_ TCID_50_/mL and 7.17 ± 0.08 log_10_ TCID_50_/mL, respectively (Figure 4D). A gradual decrease in capripoxviruses titer was observed on the subsequent days post-infection.

Control Vero cells infected with the recombinant SPPV(TKΔ)-EGFP (Figure 4E,F) were additionally monitored for GFP expression, which enabled real-time visualization of viral replication and confirmed that infection and replication in Vero cells were functional.

### 3.4. Comparison of Vero Cell Density Under Different Cultivation Methods

**Figure 5:**
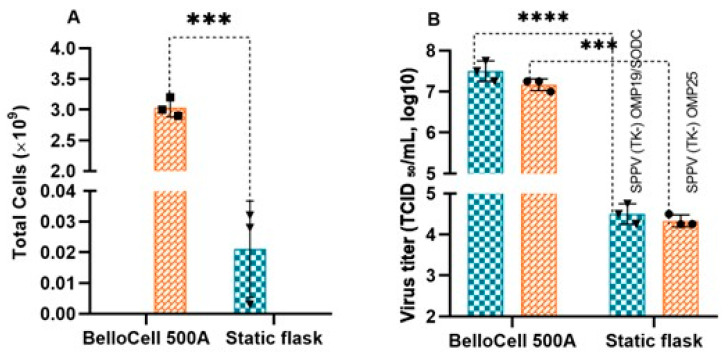
Comparative analysis of Vero cell proliferation and recombinant capripoxvirus titers in BelloCell 500A flasks and static culture flasks. (**A**) Total yields of Vero cells in BelloCell 500A compared to static flasks (*** *p* < 0.0001; *n* = 3). (**B**) Maximum titers of recombinant viruses SPPV (TK-) OMP19/SODC and SPPV (TK-) OMP25 in BelloCell 500A compared to static flasks (*** *p* < 0.0001, **** *p* < 0.001; *n* = 3).

One-day static flasks (300 cm^3^ volume) and 6-day BelloCell 500A flasks with BioNOC II^®^ macrocarriers containing Vero cell cultures were used to compare cell density. Cell dissociation was performed by washing the monolayer twice with PBS followed by the addition of 0.25% trypsin-EDTA. To determine cell concentration, the resulting suspension was mixed with 0.4% trypan blue solution at a 1:1 ratio and counted using a TC20 automated cell counter. The maximum number of cells in the static flasks was 3.2 × 10^7^, whereas the maximum number of cells in BelloCell 500A flasks with BioNOC II^®^ macrocarriers reached 3.2 × 10^9^ (Figure 5A). The number of cells obtained in BelloCell 500A flasks with BioNOC II^®^ macrocarriers was approximately 100-fold higher compared to static culture, and this difference was statistically significant (*p* < 0.001).

### 3.5. Comparison of Infectious Titers of Recombinant Capripoxviruses Cultivated by Different Methods

Comparison of virus titers obtained by different cultivation methods demonstrated that the BelloStage™-3000 bioreactor system supported significantly higher levels of recombinant virus replication compared to static flasks. The maximum titers were observed on day 4 in static flasks and on day 6 in the BelloStage™-3000 system for both recombinant strains. For the SPPV (TK-) OMP19/SODC strain, the mean titer in BelloStage™-3000 reached 7.50 log_10_ TCID_50_/mL, exceeding that of static culture (4.50 log_10_ TCID_50_/mL) by 3.00 log_10_ TCID_50_/mL (*p* < 0.0001). For the SPPV (TK-) OMP25 strain, the mean titer in BelloStage™-3000 was 7.08 log_10_ TCID_50_/mL, which was 2.75 log_10_ TCID_50_/mL higher than in static culture (4.33 log_10_ TCID_50_/mL; *p* < 0.001) (Figure 5B).

Therefore, the use of the BelloStage™-3000 bioreactor in combination with BioNOC II^®^ macrocarriers resulted in enhanced Vero cell proliferation and substantially higher recombinant capripoxvirus titers compared with conventional static flask cultures. These findings demonstrate the advantages of this scalable bioreactor-based system for efficient virus production.

## 4. Discussion

Control of viral replication is crucial, as it determines the infectious titer, which underpins quality assurance and stability in vaccine production [31]. Under scaling-up conditions, this parameter becomes critical, as it defines the reproducibility, efficacy, and industrial applicability of the production process [32,33]. In this study, serum-free production of recombinant capripoxviruses expressing *Brucella* antigens in Vero cells was investigated using the BelloStage™-3000 bioreactor with BioNOC II^®^ macrocarriers in comparison with conventional static cultivation. Cell attachment and growth, process parameter control, infectious titers, and production dynamics were evaluated, allowing the scalability and technological robustness of the bioreactor-based approach to be determined.

BelloStage™-3000 is an advanced bioreactor system designed for generating high-density cultures of animal, mammalian, and insect cells, which are used for protein expression, virus production, and monoclonal antibody generation under controlled laboratory conditions. The system’s design ensures optimal gas exchange, uniform nutrient distribution, and efficient removal of metabolic byproducts. These features create favorable conditions for the cultivation of both adherent and suspension cells, as well as for viral replication [34,35]. BioNOC II^®^ is a macroporous carrier that supports the growth of anchorage-dependent cells including animal, mammalian, and insect cells in either serum-containing or serum-free culture media. It produces higher cell yields by providing a higher surface area (up to 2.730 cm^2^/g) that allows the growth of up to 1.0 × 10^9^ cells. Due to its high porosity (50–200 μm), adherent cells are easily exposed to the necessary amount of culture media and aeration [36].

The integration of BioNOC II^®^ macrocarriers into the bioprocess provided optimal conditions for Vero cell attachment and proliferation. Using the BelloStage™-3000 system in combination with BioNOC II^®^ macrocarriers resulted in a 100-fold increase in cell concentration compared to traditional static flasks, demonstrating a substantially higher efficiency of cell growth. 

Similar results were obtained in a study by Guo et al. [30], where the BelloStage™3000 system (formerly known as CelCradle™) was used to culture Vero cells on BioNOC II^®^ macrocarriers. The authors showed that the use of this bioreactor platform was able to achieve a tenfold increase in cell concentration over the initial seeding density, indicating its high efficiency in enabling cell attachment and proliferation. Similar conclusions were drawn by Offersgaard et al. [37], who investigated the reproduction of SARS-CoV-2 virus in Vero cells grown in the BelloStage™-3000 system using the same macrocarriers. In their work, they achieved a 13-fold increase in cell concentration compared to the initial values, which confirms the reproducibility and versatility of this technology when working with different viruses.

In the studies by Rhazi et al. [38], cultivation of Vero cells in the BelloStage™-3000 system (formerly known as CelCradle™) resulted in an even more pronounced effect, with a 19-fold increase in cell numbers compared to the seeding density. These results further emphasize the potential of the BelloStage™-3000 platform for generating high-density cell cultures and its applicability in viral bioprocesses.

In the available literature, no studies have been identified that specifically investigate the reproduction of recombinant capripoxviruses in the BelloStage™-3000 system. Nevertheless, Rhazi et al. [38] demonstrated the successful application of the BelloStage™-3000 system for the cultivation of nodular dermatitis virus, a member of the capripoxvirus genus. The authors demonstrated that the viral titers obtained in BelloStage™-3000 and in the conventional cultivation system (Cell Factories) did not differ significantly (6.5 log_10_TCID_50_/mL and 6.9 log_10_TCID_50_/mL, respectively). At the same time, the cell-specific viral yield was markedly higher under BelloStage™-3000 conditions, reaching 21.9 virions per cell compared to 10 virions per cell in the static system. Comparable results were obtained by the same authors for Rift Valley fever virus and peste des petits ruminants virus, where viral titers also did not show substantial differences relative to Cell Factories. It is likely that the reduction in viral productivity was caused by nutrient limitation, which led to diminished cellular metabolic activity, decreased cell viability, and consequently lower levels of viral replication.

In contrast, multiple independent studies report that application of the BelloStage™-3000 platform markedly enhanced viral titers for Japanese encephalitis virus, hepatitis D virus, influenza virus, SARS-CoV-2, and dengue virus [30,37,38,39,40,41].

Reproduction of recombinant capripoxviruses in Vero cells grown on BioNOC II^®^ macrocarriers using the BelloStage™-3000 system was associated with efficient viral replication and a marked increase in SPPV(TK-)-OMP19/SODC and SPPV(TK-)-OMP25 titers. The differences compared to static culture were statistically significant for SPPV(TK-)-OMP19/SODC (*p* < 0.0001) and for SPPV(TK-)-OMP25 (*p* < 0.001).

To better evaluate the productivity of the two cultivation systems, we calculated the number of virions produced per cell. For cultures in the BelloCell 500A bioreactor (BelloStage™-3000), with a final harvest volume of 1500 mL and a cell density of 3.2 × 10^9^, the specific yields were approximately 14.8 virions per cell for SPPV(TK-)-OMP19/SODC and 6.9 virions per cell for SPPV(TK-)-OMP25. In contrast, in static flasks (300 mL harvest volume, 3.2 × 10^7^ cells), the calculated yields were only 0.30 and 0.20 virions per cell, respectively. Thus, the use of the BelloStage™-3000 system resulted in an approximately 50-fold and 35-fold increase in virus yield per cell for SPPV(TK-)-OMP19/SODC and SPPV(TK-)-OMP25, respectively, compared with the conventional static system. These findings clearly demonstrate the superior efficiency of the BelloStage™-3000 system for recombinant capripoxvirus production.

Genetic analysis confirmed the stable integration and maintenance of the foreign antigen-encoding sequences within the recombinant SPPV genome during cultivation. The presence of these inserts was verified by PCR (see Supplementary Material File S3, Figure S1) using gene-specific primers for each foreign sequence and universal primers targeting the thymidine kinase (TK) locus (PCR-TK-F: AATTATAGGACCTATGTTTTCTGGC; PCR-TK-R1: CAGCGTCTTTATAACATTCCAT), into which the foreign sequences had been integrated. These results provide further assurance that the recombinant viruses retained their engineered genetic properties under bioreactor conditions, which is a critical factor for vaccine development and downstream applications.

It should be noted that the BelloStage™-3000 bioreactor system is a laboratory-scale model intended for small-scale studies under controlled conditions. The data obtained on the replication of recombinant capripoxviruses are preliminary and will require further optimization for industrial applications. Future studies will focus on scaling up to production-scale systems such as TideXcell™, with the aim of improving viral yields and assessing their suitability for vaccine manufacturing.

TideXcell™ represents a linearly scalable system, ranging from laboratory to production scale. It ensures seamless technology transfer by employing the same culture principle across scales, while the TideXcell™ Harvesting System enables automated and closed harvesting and seed transfer, reducing bioprocessing time [42].

Thus, the combination of BioNOC II^®^ macrocarriers with the capabilities of the BelloStage™-3000 system enables scalable and reproducible production of recombinant capripoxviruses, markedly surpassing traditional culture methods. These findings highlight the crucial role of modern bioreactor technologies in industrial vaccinology and open promising prospects for large-scale production of vaccines not only against brucellosis but also against other viral infections.

## 5. Conclusions

In this study, we demonstrated the efficient replication of recombinant capripoxviruses in Vero cells, achieving a significant increase in viral titers compared with traditional static methods. The observed cell growth dynamics, stability of process parameters, and high virus yields highlight the successful cultivation of recombinant viruses. These results were obtained using the BelloStage™-3000 system, confirming that this bioreactor can support robust and scalable virus production. Together, the findings indicate strong potential for scaling up the production of viral vectors and recombinant vaccines under production conditions.

## Data Availability

The original contributions presented in the study are included in the article/Supplementary Material; further inquiries can be directed to the corresponding authors.

## Supplementary Materials

The following supporting information can be downloaded at https://www.mdpi.com/article/10.3390/cells14201631/s1. Figure S1. Electrophoretic profile of PCR products. M—molecular weight marker (Invitrogen 1 kb Plus); lanes 1–2: SPPV(TK-)-OMP25; lanes 3–5: SPPV(TK-)-OMP19/SODC.
