# Supplementary material for: BelloStage™-3000 Bioreactor Versus Conventional Cultivation of Recombinant Capripoxvirus Expressing Brucella Antigens in Vero Cells: A Step Towards the Development of a New Human Brucellosis Vaccine
Source: Cells. 2025 Oct 20;14(20):1631. doi: 10.3390/cells14201631 (PMC12563591; doi:10.3390/cells14201631)
Supplement: Supplementary file 1 [file cells-14-01631-s001.zip › Supplementary Materials File S1.pdf]

Supplementary Materials

Title: *Mycoplasma Detection*

Description:

This supplementary document describes the procedure used to assess the presence of mycoplasmas in cell cultures with the MycoFluor™ Mycoplasma Detection Kit (Invitrogen, Thermo Fisher Scientific, Waltham, MA, USA; Cat. #M7006), following the manufacturer's instructions.

Protocol steps:

- Cells were fixed using a fixative composed of three parts methanol and one part glycerol-acetic acid.
- Fixed samples were washed twice with distilled water to remove residual fixative.
- Cells were incubated with the fluorescent probe provided in the kit.
- After incubation, cells were washed with PBS and examined under a fluorescence microscope.
- Positive and negative controls were included in each run to ensure assay specificity.

Notes:

- This supplementary protocol provides detailed operational steps complementing the abbreviated description in the main manuscript.

Link to main manuscript: Section 2.1. "*2.1. Cell lines and culture media*).
