# Supplementary material for: BelloStage™-3000 Bioreactor Versus Conventional Cultivation of Recombinant Capripoxvirus Expressing Brucella Antigens in Vero Cells: A Step Towards the Development of a New Human Brucellosis Vaccine
Source: Cells. 2025 Oct 20;14(20):1631. doi: 10.3390/cells14201631 (PMC12563591; doi:10.3390/cells14201631)
Supplement: Supplementary file 1 [file cells-14-01631-s001.zip › Supplementary Materials File S2.pdf]

Supplementary Materials

Title: *Detailed Protocol for Cultivation of BSR and Vero Cells in the BelloStage™-3000 Bioreactor System*

Description:
This supplementary document provides the complete methodology for culturing BSR and Vero cells on BioNOC II® macrocarriers in the BelloStage™-3000 bioreactor system using OptiPRO™ SFM serum-free medium supplemented with L-glutamine, penicillin, and streptomycin.

The protocol includes:

1. *Initial cell culture*
   - BSR and Vero cells were first cultured in 300 cm^3 culture flasks (#90300, Techno Plastic Products AG, Switzerland) using OptiPRO™ SFM supplemented with L-glutamine.
2. *Cell dissociation*
   - Wash the cell monolayer twice with PBS (#10010023, Gibco™, USA).
   - Add 0.25% trypsin-EDTA (1X) (#25200056, Gibco™, USA) and incubate for 3 min at 37 °C in a CO_2 incubator.
3. *Cell preparation*
   - Centrifuge detached cells at 180 × g for 5 min and resuspend in 20 mL of fresh OptiPRO™ SFM with L-glutamine, penicillin, and streptomycin.
   - Adjust cell concentration to $\geq 1.5 \times 10^7$ cells/mL.
4. *Inoculation into macrocarriers*
   - Add 20 mL of cell suspension to BelloCell™ 500A flasks containing BioNOC II® macrocarriers pre-treated with 100 mL of medium.
   - Seal the flasks with white caps and incubate at 37 °C.
   - Manually mix the culture every 15 min during the first hour and every 30 min during the subsequent 4 h to promote cell attachment to the macrocarriers.
   - Ensure macrocarriers remain fully submerged in the cell suspension.
5. *Preparation for BelloStage™-3000 system*
   - After cell attachment, add 380 mL of fresh OptiPRO™ SFM with L-glutamine.
   - Replace the white cap with a blue cap containing a 0.22 μm filtration membrane.
6. *Cultivation in BelloStage™-3000*
   - Transfer the flasks to the BelloStage™-3000 system.
   - Culture cells under the original system parameters:
     - ✓ Lift speed: 1.0 mm/s
     - ✓ Top hold time (T_H): 10 s
     - ✓ Lowering speed: 1.0 mm/s
     - ✓ Bottom hold time (B_H): 10 s
   - Replace 60% of the medium with fresh medium after 48 h.
   - Monitor cell density, pH (7.0–7.4), and glucose concentration (≥1.0 g/L) daily.

Notes:

- Detailed operational parameters, including lift speeds, hold times, and mixing schedules, are provided in this document.
- This supplementary material complements the abbreviated methodology described in Section 2.5 of the main manuscript.

Link to main manuscript: Section 2.5 "Vero Cell Cultivation in the BelloStage™-3000 Bioreactor"
