# Supplementary material for: BelloStage™-3000 Bioreactor Versus Conventional Cultivation of Recombinant Capripoxvirus Expressing Brucella Antigens in Vero Cells: A Step Towards the Development of a New Human Brucellosis Vaccine
Source: Cells. 2025 Oct 20;14(20):1631. doi: 10.3390/cells14201631 (PMC12563591; doi:10.3390/cells14201631)
Supplement: Supplementary file 1 [file cells-14-01631-s001.zip › Supplementary Materials File S3.pdf]

Supplementary Materials

Title: *Verification of Foreign Gene Insertions in the Recombinant SPPV Genome*

Description:

The presence of foreign gene insertions in the viral genome was assessed by PCR using gene-specific primers for each insert and universal primers targeting the thymidine kinase (TK) locus (PCR-TK-F: AATTATAGGACCTATGTTTTCTGGC; PCR-TK-R1: CAGCGTCTTTATAACATTCCAT), into which the foreign sequences had been integrated.

Results:

Electrophoretic analysis confirmed the expected amplicons corresponding to the foreign sequences.

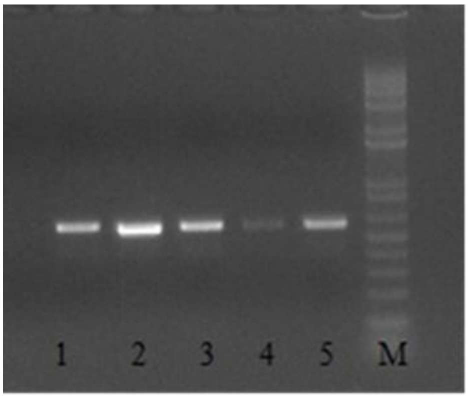

Figure S1. Electrophoretic profile of PCR products. M – molecular weight marker (Invitrogen 1 kb Plus); lanes 1–2: SPPV(TK−)-OMP25; lanes 3–5: SPPV(TK−)-OMP19/SODC
